# Crystal structure of bis­(bis­{μ_3_-3-methyl-3-[(4-nitro-2-oxido­benzyl­idene)amino]­propane-1,3-diolato}tris­[chlorido­(dimethyl sulfoxide)­iron(III)]) dimethyl sulfoxide hepta­solvate dihydrate

**DOI:** 10.1107/S2056989016018508

**Published:** 2016-11-29

**Authors:** Eduard Chygorin, Yuri Smal, Irina V. Omelchenko

**Affiliations:** a Department of Inorganic Chemistry, Taras Shevchenko National University of Kyiv, 64 Volodymyrs’ka St., Kyiv 01601, Ukraine; b SSI ‘Institute for Single Crystals’ NAS of Ukraine, 60 Nauky ave, Kharkiv, 61072, Ukraine

**Keywords:** crystal structure, trinuclear, iron(III), Schiff base ligand

## Abstract

The title compound, [Fe_3_(C_11_H_11_N_2_O_5_)_2_Cl_3_(C_2_H_6_OS)_3_]_2_·7C_2_H_6_OS·2H_2_O, was isolated accidentally from an Fe^0^–NiCl_2_·6H_2_O–H_3_ *L*–TEA–DMSO system [where H_3_ *L* is the product of the condensation between *p*-nitro­salicyl­aldehyde and 2-amino-2-methyl­propane-1,3-diol and dimethyl sulfoxide (DMSO), and TEA is triethylamine]. The structure is based on a trinuclear {Fe_3_(μ-O)_4_} core, with an angular arrangement of the Fe^III^ ions that can be explained by the geometrical restrictions of two bulky ligands, each coordinating to all of the metal cations.

## Abstract 2

The title compound is based on a trinuclear {Fe_3_(μ-O)_4_} core with an angular arrangement of the Fe^III^ ions that can be explained by geometrical restrictions of two bulky ligands each coordinated to all the metal centres.

## Chemical context   

Almost 30% of GDP (gross domestic product) is generated through catalysis, which explains the ongoing inter­est in the development of compounds with potential as new efficient catalysts. Polynuclear associates have been found to be co-factors of many enzymes and catalysts for various pro­cesses (Buchwalter *et al.*, 2015 ▸). In this work, we present the synthesis of a new trinuclear Fe^III^ complex obtained accidentally while exploring the Fe^0^–NiCl_2_·6H_2_O–H_3_ *L*–TEA–DMSO system (TEA is tri­ethyl­amine and DMSO is dimethyl sulfoxide). We did not investigate this complex for any catalytic activity, although it has a hypothetical practical inter­est because it was obtained in facile way from commercially abundant air-stable non-haza­rdous materials and consists of redoxactive metal atoms and ligands. The synthesis is based on the self-assembling paradigm, in particular on direct synthesis (Garnovskii *et al.*, 1999 ▸); the metal ions and ligands are allowed to choose the most favourable charge and coordination modes and do not require specific synthetic manipulations and laboratory equipment. However, under these conditions we cannot predict the structure of the final mol­ecule that will be obtained. Earlier, our group has shown the successful application of this approach for obtaining novel monometallic [either polynuclear, as in Babich & Kokozay (1997 ▸), or mixed valence, as in Kovbasyuk *et al.* (1997 ▸)], heterobimetallic [either polynuclear, as in Kovbasyuk *et al.* (1998 ▸), Vassilyeva *et al.* (1997 ▸) and Nikitina *et al.* (2008 ▸) or polymeric, as in Nesterova *et al.* (2004 ▸, 2005 ▸, 2008 ▸)] and heterotrimetallic [as in Nesterov *et al.* (2011 ▸)] complexes.

## Structural commentary   

**Figure 1:**
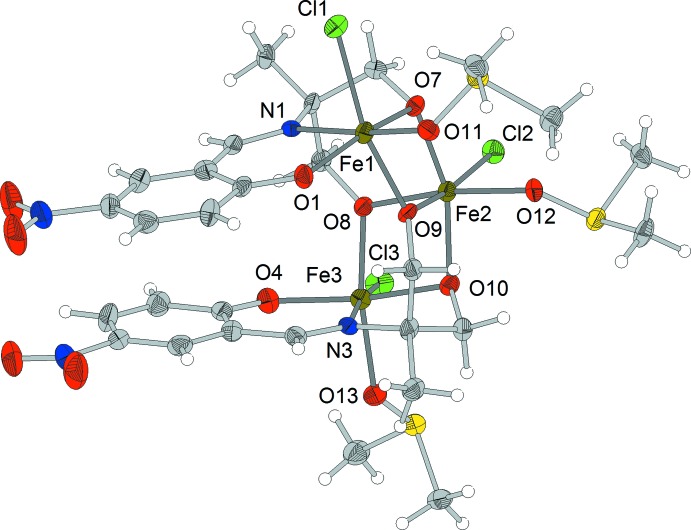
The mol­ecular structure of the title complex. Displacement ellipsoids are drawn at the 70% probability level. Colour key: Fe dark green, N blue, O red, S yellow and Cl green.

The mol­ecular complex [Fe^III^_3_ *L*_2_Cl_3_(DMSO)_3_]_2_·7DMSO·2H_2_O is based on a trinuclear {Fe_3_(μ-O)_4_} core with an angular arrangement of the metal cations [the Fe⋯Fe⋯Fe angle is 104.70 (4)°], linked pairwise by two μ-O bridges from the fully deprotonated Schiff base ligand (Fig. 1 ▸). The structure can also be viewed as a combination of two {Fe^III^ *L*} blocks joined through a central Fe^III^ ion *via* alk­oxy bridges and completed by chloride ligands and solvent mol­ecules (DMSO and water).
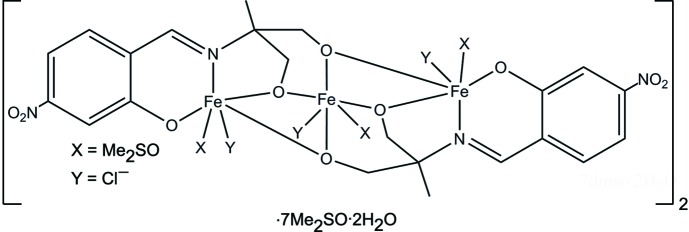


The {Fe(μ-O)_2_Fe} fragments are almost perpendicular [angle between planes = 96.4 (1)°]. Both Schiff base ligands reveal a 3.2211 coordination mode (Coxall *et al.*, 2000 ▸). The NO_4_Cl donor set of each of the terminal Fe^III^ cations includes two μ-O-bridging atoms from alk­oxy groups, as well as N and O atoms from the Schiff base ligands. The O_5_Cl donor set of the central Fe^III^ atom includes four μ-O-bridging atoms from the alk­oxy groups of two ligands. Both donor sets contain one O atom from a coordinating DMSO mol­ecule and one chloride ligand. All three Fe^II^ atoms have a distorted octa­hedral environment. The main source of distortion is the difference between the Fe—Cl [2.332 (2)–2.378 (2) Å], Fe—O [1.925 (3)–2.046 (5) Å] and Fe—N [2.132 (6)-2.157 (4) Å] bond lengths. The deviations of the O(N)—Fe—O(N,Cl) bond angles from ideal octa­hedral values are up to 19.4 (2)°, the mean deviation being slightly higher for the terminal complex fragments than for the central one [8.54 (5) *versus* 7.68 (5)°]. It should be noted that the coordination environments of the terminal metal cations are not equivalent. The N atom occupies an axial position at atom Fe3, but an equatorial one at atom Fe1, assuming the chloride ligand is in an axial position in both polyhedra, due to an anti­parallel arrangement of the two Schiff base ligands, which is also favourable for an intra­molecular stacking inter­action between the benzene rings [inter­centroid distance = 4.034 (4) Å, plane-to-centroid dis­tance = 3.505 (7) Å, centroid displacement = 2.00 (1) Å and angle between planes = 7.8 (2)°]. The weak intra­molecular attractive inter­action C23—H23*C*⋯O12 (H⋯O = 2.43 Å) stabilizes the orientation of adjacent DMSO ligands.

## Supra­molecular features   

**Figure 2:**
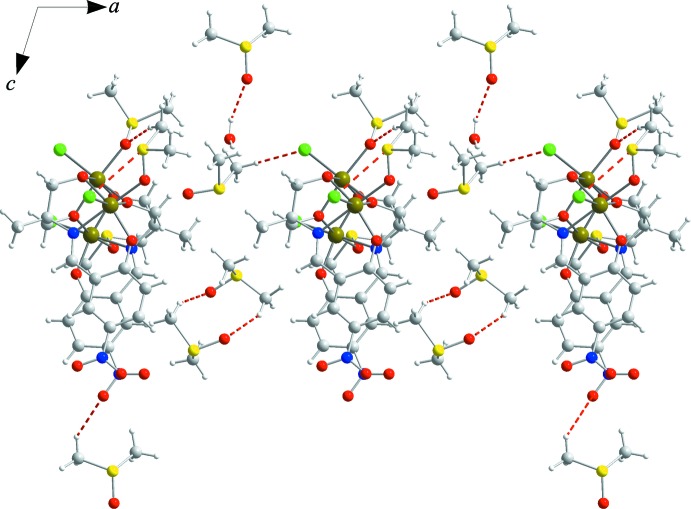
Crystal packing diagram showing the presence of supra­molecular four-membered hydrogen-bonded rings aggregating two water mol­ecules with two uncoordinated DMSO mol­ecules. Hydrogen bonds are denoted with dashed lines and H atoms have been omited for clarity.

In the crystal, there are supra­molecular four-membered hydrogen-bonded rings aggregating water mol­ecules with two non-coordinating DMSO mol­ecules (Table 1 ▸). They are linked to the mol­ecular complexes and other solvent mol­ecules by a number of weak attractive H⋯Cl, H⋯O, H⋯S, S⋯Cl and S⋯S contacts giving a three-dimensional structure (Fig. 2 ▸).

## Database survey   

A search of the Cambridge Structural Database (Version 5.37; last update March 2016; Groom *et al.*, 2016 ▸) for related com­plexes with a similar trinuclear {Me_3_(μ-*X*)_4_} core containing hexa­coordinated metal cations gave 263 hits. Though most of these cores reveal a linear arrangement of the metal atoms (207 complexes in 192 structures with an *M*—*M*—*M* angle in the range 167–180°), there are 28 strongly folded cores (82–112°) and 43 less folded cores (118–162°). Among them, three structures with the {Fe_3_(μ-O)_4_} core were found (Lieberman *et al.*, 2015 ▸), all with an angular arrangement of the Fe atoms (109–111°). There are 79 organometallic complexes based on the 2-[(2-hy­droxy­benzyl­idene)­amino]-2-methyl­propane-1,3-diol Schiff base ligand with different substituents in the benzene ring. Among them, 16 have a similar {Me_3_(μ-*X*)_4_} trinuclear core, each containing an octa­coordinated central lanthanide cation.

## Synthesis and crystallization   

To a mixture of *p*-nitro­salicylaldehyde (0.42 g, 2.5 mmol), 2-amino-2-methyl­propane-1,3-diol (0.26 g, 2.5 mmol) and tri­ethyl­amine (TEA; 0.35 ml, 2.5 mmol) in dimethyl sulfoxide (DMSO; 20 ml) were added iron powder (0.07 g, 1.25 mmol) and NiCl_2_·6H_2_O (0.3 g, 1.25 mmol) in one portion at 323–333 K and the resulting solution was stirred for 1 h to form a dark-red solution. Dark-red crystals suitable for X-ray analysis were isolated by adding Et_2_O after 2 d (yield: 0.57 g, 53%). The compound is sparingly soluble in MeOH, DMSO and DMF, and it is stable in air.

## Refinement   

Crystal data, data collection and structure refinement details are summarized in Table 2 ▸. All H atoms were placed in idealized positions (C—H = 0.95–0.99 Å and O—H = 0.87 Å) and constrained to ride on their parent atoms, with *U*_iso_(H) = 1.5*U*_eq_(C,O) for water molecules and methyl groups, and 1.2*U*_eq_(C) otherwise. Two of the non-coordinating DMSO solvent mol­ecules were disordered, each over two sites. The refined occupancy factors for the S6*A*/S6*B* disordered DMSO mol­ecule converged to 0.745:0.255. For the S7 disordered mol­ecule, the occupancy factors were fixed at 0.50:0.50 due to symmetry restrictions; two sites of this mol­ecule are located in neighbouring asymmetric parts of the unit cells and are connected by the symmetry transformation (−*x* + 1, −*y* + 2, −*z* + 1). SAME and RIGU restraints (*SHELXL2014*; Sheldrick, 2015 ▸) were applied to the atoms of all non-coordinating DMSO mol­ecules.

## Supplementary Material

Crystal structure: contains datablock(s) I. DOI: 10.1107/S2056989016018508/bg2596sup1.cif

Structure factors: contains datablock(s) I. DOI: 10.1107/S2056989016018508/bg2596Isup2.hkl

CCDC reference: 1517947

Additional supporting information: crystallographic information; 3D view; checkCIF report

## Figures and Tables

**Table 1:** Hydrogen-bond geometry (Å, °)

*D*—H⋯*A*	*D*—H	H⋯*A*	*D*⋯*A*	*D*—H⋯*A*
C30—H30*A*⋯Cl2^i^	0.98	2.75	3.689 (7)	161
C32—H32*A*⋯O6	0.98	2.60	3.397 (9)	138
C32—H32*B*⋯Cl1^ii^	0.98	2.71	3.625 (6)	155
C32—H32*C*⋯Cl3^iii^	0.98	2.82	3.659 (7)	144
C34*A*—H34*A*⋯O4^iv^	0.98	2.52	3.47 (2)	164
C33*B*—H33*D*⋯O2^v^	0.98	2.35	3.06 (3)	128
C33*B*—H33*D*⋯N2^v^	0.98	2.26	3.08 (3)	141
C34*B*—H34*D*⋯O2^v^	0.98	2.58	3.19 (4)	120
C3—H3⋯O16*A*	0.95	2.60	3.391 (9)	141
C7—H7⋯O3^ii^	0.95	2.40	3.325 (8)	164
C23—H23*C*⋯O12	0.98	2.43	3.377 (8)	163
C25—H25*A*⋯Cl2	0.98	2.80	3.524 (6)	131
C25—H25*B*⋯O10^vi^	0.98	2.39	3.303 (7)	155
C26—H26*C*⋯O1*W*	0.98	2.55	3.393 (10)	144
C27—H27*A*⋯O14	0.98	2.27	3.243 (8)	171
O1*W*—H1*WA*⋯O15^vii^	0.87	2.12	2.949 (8)	158

Symmetry codes: (i) 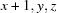; (ii) 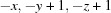; (iii) 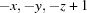; (iv) 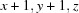; (v) 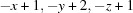; (vi) 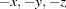; (vii) 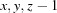.

**Table 2:** Experimental details

Crystal data	
Chemical formula	[Fe_3_(C_11_H_11_N_2_O_5_)_2_Cl_3_(C_2_H_6_OS)_3_]_2_·7C_2_H_6_OS·2H_2_O
*M*_r_	2604.36
Crystal system, space group	Triclinic, *P* 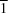
Temperature (K)	100
*a*, *b*, *c* (Å)	11.4286 (7), 12.7227 (8), 20.1915 (12)
α, β, γ (°)	94.005 (5), 105.839 (6), 103.952 (6)
*V* (Å^3^)	2712.0 (3)
*Z*	1
Radiation type	Mo *K*α
μ (mm^−1^)	1.26
Crystal size (mm)	0.4 × 0.4 × 0.4

Data collection	
Diffractometer	Agilent Xcalibur Sapphire3
Absorption correction	Multi-scan (*CrysAlis PRO*; Agilent, 2012 ▸)
*T*_min_, *T*_max_	0.985, 1.000
No. of measured, independent and observed [*I* > 2σ(*I*)] reflections	21211, 12221, 6081
*R*_int_	0.085
(sin θ/λ)_max_ (Å^−1^)	0.682

Refinement	
*R*[*F* ^2^ > 2σ(*F* ^2^)], *wR*(*F* ^2^), *S*	0.081, 0.163, 0.99
No. of reflections	12221
No. of parameters	697
No. of restraints	150
H-atom treatment	H-atom parameters constrained
Δρ_max_, Δρ_min_ (e Å^−3^)	0.96, −0.79

Computer programs: *CrysAlis PRO* (Agilent, 2012 ▸), *SHELXS97* (Sheldrick, 2008 ▸), *SHELXL2014* (Sheldrick, 2015 ▸) and *OLEX2* (Dolomanov *et al.*, 2009 ▸).

## supplementary crystallographic information

### Crystal data

**Table:**

[Fe_3_(C_11_H_11_N_2_O_5_)_2_Cl_3_(C_2_H_6_OS)_3_]_2_·7C_2_H_6_OS·2H_2_O	*Z* = 1
*M**_r_* = 2604.36	*F*(000) = 1348
Triclinic, *P*1	*D*_x_ = 1.595 Mg m^−^^3^
*a* = 11.4286 (7) Å	Mo *K*α radiation, λ = 0.71073 Å
*b* = 12.7227 (8) Å	Cell parameters from 2112 reflections
*c* = 20.1915 (12) Å	θ = 2.8–28.9°
α = 94.005 (5)°	µ = 1.26 mm^−^^1^
β = 105.839 (6)°	*T* = 100 K
γ = 103.952 (6)°	Block, metallic dark red
*V* = 2712.0 (3) Å^3^	0.4 × 0.4 × 0.4 mm

### Data collection

**Table:**

Agilent Xcalibur Sapphire3 diffractometer	12221 independent reflections
Radiation source: Enhance (Mo) X-ray Source	6081 reflections with *I* > 2σ(*I*)
Graphite monochromator	*R*_int_ = 0.085
Detector resolution: 16.1827 pixels mm^-1^	θ_max_ = 29.0°, θ_min_ = 2.8°
ω scans	*h* = −11→15
Absorption correction: multi-scan (CrysAlis PRO; Agilent, 2012)	*k* = −14→15
*T*_min_ = 0.985, *T*_max_ = 1.000	*l* = −26→27
21211 measured reflections	

### Refinement

**Table:**

Refinement on *F*^2^	150 restraints
Least-squares matrix: full	Hydrogen site location: mixed
*R*[*F*^2^ > 2σ(*F*^2^)] = 0.081	H-atom parameters constrained
*wR*(*F*^2^) = 0.163	*w* = 1/[σ^2^(*F*_o_^2^) + (0.0472*P*)^2^] where *P* = (*F*_o_^2^ + 2*F*_c_^2^)/3
*S* = 0.99	(Δ/σ)_max_ < 0.001
12221 reflections	Δρ_max_ = 0.96 e Å^−^^3^
697 parameters	Δρ_min_ = −0.79 e Å^−^^3^

### Special details

**Table:**

Geometry. All esds (except the esd in the dihedral angle between two l.s. planes) are estimated using the full covariance matrix. The cell esds are taken into account individually in the estimation of esds in distances, angles and torsion angles; correlations between esds in cell parameters are only used when they are defined by crystal symmetry. An approximate (isotropic) treatment of cell esds is used for estimating esds involving l.s. planes.

### Fractional atomic coordinates and isotropic or equivalent isotropic displacement parameters (Å^2^)

**Table:**

	*x*	*y*	*z*	*U*_iso_*/*U*_eq_	Occ. (<1)
S4	0.51353 (18)	0.00966 (16)	0.15206 (10)	0.0340 (5)	
O14	0.3925 (4)	−0.0478 (4)	0.1641 (3)	0.0435 (15)	
C29	0.4752 (7)	0.0883 (6)	0.0847 (3)	0.0354 (19)	
H29A	0.5490	0.1152	0.0686	0.053*	
H29B	0.4501	0.1506	0.1022	0.053*	
H29C	0.4051	0.0427	0.0459	0.053*	
C30	0.5565 (7)	−0.0887 (5)	0.1037 (4)	0.042 (2)	
H30A	0.6327	−0.0531	0.0918	0.062*	
H30B	0.4873	−0.1209	0.0610	0.062*	
H30C	0.5732	−0.1463	0.1317	0.062*	
S5	0.43965 (17)	0.41956 (16)	0.81772 (10)	0.0333 (5)	
O15	0.4863 (5)	0.4117 (5)	0.8934 (2)	0.0581 (18)	
C31	0.5097 (6)	0.3396 (5)	0.7736 (4)	0.0304 (18)	
H31A	0.4672	0.3292	0.7234	0.046*	
H31B	0.5012	0.2681	0.7898	0.046*	
H31C	0.5994	0.3770	0.7829	0.046*	
C32	0.2822 (5)	0.3337 (6)	0.7873 (4)	0.0342 (19)	
H32A	0.2491	0.3318	0.7369	0.051*	
H32B	0.2292	0.3625	0.8105	0.051*	
H32C	0.2816	0.2595	0.7976	0.051*	
S6A	0.6950 (3)	0.8852 (3)	0.3566 (2)	0.0711 (14)	0.745 (5)
O16A	0.6098 (8)	0.8336 (7)	0.3963 (5)	0.082 (3)	0.745 (5)
C33A	0.652 (2)	1.0034 (10)	0.3327 (7)	0.063 (4)	0.745 (5)
H33A	0.7139	1.0463	0.3128	0.094*	0.745 (5)
H33B	0.6492	1.0474	0.3738	0.094*	0.745 (5)
H33C	0.5682	0.9827	0.2980	0.094*	0.745 (5)
C34A	0.8471 (9)	0.9506 (14)	0.4144 (10)	0.080 (6)	0.745 (5)
H34A	0.8978	0.9960	0.3894	0.120*	0.745 (5)
H34B	0.8886	0.8952	0.4327	0.120*	0.745 (5)
H34C	0.8392	0.9968	0.4529	0.120*	0.745 (5)
S6B	0.7275 (11)	0.9895 (10)	0.4285 (6)	0.077 (4)	0.255 (5)
O16B	0.836 (3)	0.947 (4)	0.423 (3)	0.132 (16)	0.255 (5)
C33B	0.784 (3)	1.1108 (17)	0.4868 (13)	0.055 (8)	0.255 (5)
H33D	0.7968	1.1733	0.4613	0.083*	0.255 (5)
H33E	0.8646	1.1113	0.5202	0.083*	0.255 (5)
H33F	0.7227	1.1162	0.5117	0.083*	0.255 (5)
C34B	0.679 (6)	1.043 (3)	0.3497 (13)	0.081 (15)	0.255 (5)
H34D	0.6079	1.0733	0.3500	0.121*	0.255 (5)
H34E	0.6520	0.9839	0.3103	0.121*	0.255 (5)
H34F	0.7493	1.1004	0.3452	0.121*	0.255 (5)
S7	0.6166 (5)	1.0553 (4)	0.5331 (2)	0.0476 (12)	0.5
O17	0.7281 (9)	1.0973 (8)	0.5074 (6)	0.050 (3)	0.5
C35	0.4830 (11)	1.0142 (15)	0.4589 (6)	0.059 (5)	0.5
H35A	0.4067	0.9901	0.4734	0.088*	0.5
H35B	0.4909	0.9537	0.4292	0.088*	0.5
H35C	0.4770	1.0760	0.4330	0.088*	0.5
C36	0.6246 (14)	0.9233 (8)	0.5537 (8)	0.041 (4)	0.5
H36A	0.5472	0.8864	0.5641	0.062*	0.5
H36B	0.6978	0.9304	0.5943	0.062*	0.5
H36C	0.6331	0.8802	0.5140	0.062*	0.5
Fe1	0.08933 (9)	0.47702 (8)	0.19107 (5)	0.0185 (2)	
Fe2	0.00286 (9)	0.22654 (8)	0.13027 (5)	0.0177 (2)	
Fe3	0.04940 (9)	0.10574 (8)	0.26158 (5)	0.0197 (2)	
Cl1	−0.00668 (16)	0.62291 (13)	0.17118 (9)	0.0241 (4)	
Cl2	−0.18724 (15)	0.10406 (13)	0.06019 (9)	0.0228 (4)	
Cl3	−0.13256 (16)	−0.04056 (14)	0.22910 (10)	0.0294 (4)	
S1	0.15125 (16)	0.57248 (14)	0.05967 (9)	0.0218 (4)	
S2	0.07383 (17)	0.13945 (14)	−0.00193 (9)	0.0228 (4)	
S3	0.11479 (17)	−0.12355 (14)	0.26358 (9)	0.0248 (4)	
O1	0.2222 (4)	0.5484 (3)	0.2747 (2)	0.0232 (11)	
O2	0.3496 (5)	0.7307 (5)	0.5881 (3)	0.0513 (17)	
O3	0.1605 (5)	0.6284 (5)	0.5700 (3)	0.0490 (17)	
O4	0.0384 (4)	0.1516 (4)	0.3533 (2)	0.0246 (11)	
O5	0.4440 (5)	0.5034 (5)	0.5888 (3)	0.0509 (17)	
O6	0.3049 (5)	0.4414 (4)	0.6405 (3)	0.0499 (16)	
O7	−0.0604 (4)	0.3623 (3)	0.1237 (2)	0.0171 (10)	
O8	−0.0458 (4)	0.2050 (3)	0.2166 (2)	0.0182 (10)	
O9	0.1544 (4)	0.3463 (3)	0.1846 (2)	0.0179 (10)	
O10	0.0923 (4)	0.1161 (3)	0.1704 (2)	0.0196 (11)	
O11	0.1978 (4)	0.5401 (4)	0.1319 (2)	0.0229 (11)	
O12	0.0731 (4)	0.2370 (3)	0.0472 (2)	0.0196 (11)	
O13	0.1561 (4)	−0.0003 (3)	0.2918 (2)	0.0243 (11)	
N1	−0.0261 (5)	0.4270 (4)	0.2565 (3)	0.0152 (12)	
N2	0.2514 (6)	0.6657 (5)	0.5500 (3)	0.0312 (15)	
N3	0.2315 (5)	0.2249 (4)	0.2927 (3)	0.0186 (13)	
N4	0.3423 (6)	0.4435 (5)	0.5889 (3)	0.0370 (17)	
C1	0.1212 (6)	0.5323 (5)	0.3645 (3)	0.0201 (16)	
C2	0.2279 (6)	0.5727 (5)	0.3398 (3)	0.0200 (16)	
C3	0.3396 (6)	0.6422 (5)	0.3870 (4)	0.0256 (18)	
H3	0.4110	0.6684	0.3712	0.031*	
C4	0.3478 (7)	0.6724 (5)	0.4541 (4)	0.0269 (17)	
H4	0.4235	0.7204	0.4846	0.032*	
C5	0.2436 (7)	0.6321 (5)	0.4784 (4)	0.0242 (17)	
C6	0.1330 (6)	0.5636 (5)	0.4342 (3)	0.0237 (17)	
H6	0.0636	0.5373	0.4515	0.028*	
C7	0.0034 (6)	0.4584 (5)	0.3219 (3)	0.0196 (16)	
H7	−0.0578	0.4304	0.3446	0.023*	
C8	0.2326 (6)	0.2906 (6)	0.4096 (4)	0.0238 (17)	
C9	0.1100 (7)	0.2252 (6)	0.4059 (4)	0.0233 (16)	
C10	0.0664 (7)	0.2414 (6)	0.4638 (4)	0.0276 (18)	
H10	−0.0163	0.2012	0.4621	0.033*	
C11	0.1388 (7)	0.3125 (6)	0.5218 (4)	0.034 (2)	
H11	0.1064	0.3218	0.5598	0.040*	
C12	0.2624 (7)	0.3730 (6)	0.5260 (4)	0.0297 (18)	
C13	0.3071 (6)	0.3625 (5)	0.4698 (4)	0.0248 (17)	
H13	0.3893	0.4046	0.4721	0.030*	
C14	0.2859 (6)	0.2869 (5)	0.3512 (3)	0.0229 (16)	
H14	0.3674	0.3344	0.3575	0.027*	
C15	−0.1529 (6)	0.3491 (5)	0.2202 (3)	0.0174 (15)	
C16	−0.1743 (6)	0.3578 (5)	0.1428 (3)	0.0178 (15)	
H16A	−0.2424	0.2937	0.1148	0.021*	
H16B	−0.2017	0.4245	0.1324	0.021*	
C17	−0.1451 (6)	0.2349 (5)	0.2352 (3)	0.0201 (16)	
H17A	−0.1327	0.2319	0.2855	0.024*	
H17B	−0.2262	0.1812	0.2092	0.024*	
C18	−0.2612 (6)	0.3752 (5)	0.2418 (3)	0.0203 (16)	
H18A	−0.2616	0.3503	0.2866	0.031*	
H18B	−0.3414	0.3376	0.2065	0.031*	
H18C	−0.2506	0.4544	0.2462	0.031*	
C19	0.2947 (6)	0.2383 (5)	0.2368 (3)	0.0197 (16)	
C20	0.2822 (6)	0.3438 (5)	0.2084 (3)	0.0218 (16)	
H20A	0.3303	0.4067	0.2454	0.026*	
H20B	0.3194	0.3512	0.1695	0.026*	
C21	0.2246 (6)	0.1391 (5)	0.1814 (3)	0.0199 (16)	
H21A	0.2435	0.1532	0.1373	0.024*	
H21B	0.2538	0.0748	0.1962	0.024*	
C22	0.4351 (6)	0.2406 (6)	0.2638 (4)	0.0289 (18)	
H22A	0.4833	0.3114	0.2922	0.043*	
H22B	0.4673	0.2300	0.2242	0.043*	
H22C	0.4439	0.1818	0.2921	0.043*	
C23	0.1973 (6)	0.4836 (5)	0.0061 (4)	0.0261 (17)	
H23A	0.2890	0.4950	0.0231	0.039*	
H23B	0.1737	0.4992	−0.0419	0.039*	
H23C	0.1546	0.4074	0.0075	0.039*	
C24	0.2629 (6)	0.6974 (5)	0.0616 (4)	0.0287 (18)	
H24A	0.2630	0.7521	0.0982	0.043*	
H24B	0.2403	0.7234	0.0165	0.043*	
H24C	0.3473	0.6856	0.0711	0.043*	
C25	−0.0546 (6)	0.1214 (5)	−0.0784 (3)	0.0247 (17)	
H25A	−0.1343	0.0971	−0.0673	0.037*	
H25B	−0.0501	0.0660	−0.1132	0.037*	
H25C	−0.0504	0.1909	−0.0969	0.037*	
C26	0.1979 (6)	0.1938 (6)	−0.0382 (4)	0.0309 (19)	
H26A	0.1785	0.2534	−0.0637	0.046*	
H26B	0.2055	0.1360	−0.0702	0.046*	
H26C	0.2777	0.2220	−0.0008	0.046*	
C27	0.2614 (6)	−0.1576 (6)	0.2754 (4)	0.0292 (18)	
H27A	0.3073	−0.1184	0.2461	0.044*	
H27B	0.2449	−0.2367	0.2624	0.044*	
H27C	0.3124	−0.1365	0.3244	0.044*	
C28	0.0646 (7)	−0.1885 (6)	0.3300 (4)	0.0311 (19)	
H28A	0.1301	−0.1611	0.3748	0.047*	
H28B	0.0498	−0.2678	0.3194	0.047*	
H28C	−0.0139	−0.1723	0.3324	0.047*	
O1W	0.4926 (8)	0.3634 (5)	0.0349 (4)	0.077 (2)	
H1WA	0.4820	0.3589	−0.0097	0.116*	
H1WB	0.5064	0.4313	0.0524	0.116*	

### Atomic displacement parameters (Å^2^)

**Table:**

	*U*^11^	*U*^22^	*U*^33^	*U*^12^	*U*^13^	*U*^23^
S4	0.0300 (11)	0.0397 (13)	0.0301 (12)	0.0055 (9)	0.0090 (10)	0.0044 (9)
O14	0.038 (3)	0.047 (4)	0.052 (4)	0.006 (3)	0.029 (3)	0.006 (3)
C29	0.034 (4)	0.034 (5)	0.042 (5)	0.012 (4)	0.016 (4)	0.001 (4)
C30	0.036 (5)	0.044 (5)	0.053 (6)	0.009 (4)	0.028 (5)	0.014 (4)
S5	0.0225 (10)	0.0383 (13)	0.0352 (12)	0.0077 (9)	0.0043 (10)	−0.0013 (10)
O15	0.041 (3)	0.108 (5)	0.033 (3)	0.036 (3)	0.012 (3)	−0.001 (3)
C31	0.024 (4)	0.039 (5)	0.026 (4)	0.009 (3)	0.005 (3)	0.005 (3)
C32	0.025 (4)	0.033 (5)	0.046 (5)	0.008 (3)	0.015 (4)	0.003 (4)
S6A	0.050 (2)	0.041 (2)	0.105 (3)	−0.0100 (16)	0.025 (2)	−0.021 (2)
O16A	0.055 (6)	0.063 (6)	0.119 (8)	−0.005 (5)	0.034 (6)	0.002 (5)
C33A	0.071 (11)	0.079 (9)	0.041 (8)	0.023 (8)	0.022 (8)	0.003 (7)
C34A	0.057 (8)	0.072 (11)	0.100 (12)	−0.006 (7)	0.019 (7)	0.042 (9)
S6B	0.085 (9)	0.102 (10)	0.057 (7)	0.026 (7)	0.038 (7)	0.029 (6)
O16B	0.12 (2)	0.17 (3)	0.13 (3)	0.07 (2)	0.044 (18)	0.03 (2)
C33B	0.033 (18)	0.095 (16)	0.047 (14)	0.019 (12)	0.017 (13)	0.047 (11)
C34B	0.08 (3)	0.11 (3)	0.056 (14)	0.02 (3)	0.028 (15)	0.027 (15)
S7	0.063 (3)	0.046 (3)	0.032 (3)	0.019 (2)	0.009 (2)	−0.002 (2)
O17	0.056 (7)	0.031 (7)	0.053 (8)	0.002 (5)	0.006 (6)	0.014 (6)
C35	0.057 (9)	0.076 (13)	0.042 (9)	0.019 (8)	0.013 (7)	0.004 (8)
C36	0.041 (9)	0.034 (7)	0.040 (9)	0.001 (6)	0.009 (8)	−0.003 (6)
Fe1	0.0180 (5)	0.0187 (6)	0.0194 (6)	0.0032 (4)	0.0085 (5)	0.0004 (4)
Fe2	0.0165 (5)	0.0193 (6)	0.0171 (5)	0.0040 (4)	0.0057 (4)	0.0007 (4)
Fe3	0.0193 (5)	0.0196 (6)	0.0199 (6)	0.0047 (4)	0.0059 (5)	0.0027 (4)
Cl1	0.0243 (9)	0.0198 (10)	0.0303 (10)	0.0068 (7)	0.0114 (8)	0.0020 (8)
Cl2	0.0194 (9)	0.0228 (10)	0.0226 (10)	0.0017 (7)	0.0047 (8)	−0.0002 (7)
Cl3	0.0239 (10)	0.0238 (11)	0.0374 (12)	0.0022 (8)	0.0078 (9)	0.0045 (8)
S1	0.0176 (9)	0.0273 (11)	0.0211 (10)	0.0054 (7)	0.0070 (8)	0.0044 (8)
S2	0.0278 (10)	0.0219 (10)	0.0226 (10)	0.0094 (8)	0.0113 (9)	0.0024 (8)
S3	0.0275 (10)	0.0239 (11)	0.0242 (11)	0.0096 (8)	0.0075 (9)	0.0038 (8)
O1	0.020 (3)	0.028 (3)	0.019 (3)	0.004 (2)	0.005 (2)	−0.004 (2)
O2	0.044 (4)	0.068 (4)	0.021 (3)	−0.006 (3)	0.000 (3)	−0.017 (3)
O3	0.039 (3)	0.077 (5)	0.025 (3)	0.000 (3)	0.014 (3)	−0.002 (3)
O4	0.023 (3)	0.025 (3)	0.024 (3)	0.002 (2)	0.009 (2)	0.004 (2)
O5	0.049 (4)	0.058 (4)	0.035 (4)	0.007 (3)	0.008 (3)	−0.017 (3)
O6	0.060 (4)	0.064 (4)	0.023 (3)	0.019 (3)	0.009 (3)	−0.008 (3)
O7	0.018 (2)	0.016 (3)	0.020 (3)	0.0043 (19)	0.011 (2)	0.002 (2)
O8	0.017 (2)	0.020 (3)	0.019 (3)	0.007 (2)	0.006 (2)	0.002 (2)
O9	0.018 (2)	0.016 (3)	0.021 (3)	0.0069 (19)	0.007 (2)	−0.001 (2)
O10	0.016 (2)	0.022 (3)	0.022 (3)	0.005 (2)	0.008 (2)	0.001 (2)
O11	0.018 (2)	0.031 (3)	0.022 (3)	0.009 (2)	0.009 (2)	0.008 (2)
O12	0.023 (3)	0.019 (3)	0.018 (3)	0.004 (2)	0.009 (2)	0.002 (2)
O13	0.033 (3)	0.015 (3)	0.026 (3)	0.011 (2)	0.008 (2)	0.003 (2)
N1	0.012 (3)	0.016 (3)	0.017 (3)	0.005 (2)	0.002 (3)	0.002 (2)
N2	0.034 (4)	0.037 (4)	0.019 (4)	0.009 (3)	0.003 (3)	0.000 (3)
N3	0.015 (3)	0.022 (3)	0.017 (3)	0.006 (2)	0.001 (3)	0.000 (3)
N4	0.044 (4)	0.047 (5)	0.016 (4)	0.019 (4)	−0.002 (3)	−0.005 (3)
C1	0.022 (4)	0.016 (4)	0.023 (4)	0.003 (3)	0.008 (3)	0.004 (3)
C2	0.025 (4)	0.015 (4)	0.020 (4)	0.007 (3)	0.007 (3)	−0.001 (3)
C3	0.029 (4)	0.022 (4)	0.024 (4)	0.003 (3)	0.011 (4)	−0.004 (3)
C4	0.024 (4)	0.026 (4)	0.028 (4)	0.008 (3)	0.004 (4)	0.003 (3)
C5	0.034 (4)	0.025 (4)	0.018 (4)	0.010 (3)	0.011 (4)	0.004 (3)
C6	0.023 (4)	0.025 (4)	0.023 (4)	0.007 (3)	0.007 (3)	0.000 (3)
C7	0.022 (4)	0.023 (4)	0.019 (4)	0.012 (3)	0.009 (3)	0.005 (3)
C8	0.023 (4)	0.028 (4)	0.021 (4)	0.009 (3)	0.005 (3)	0.002 (3)
C9	0.029 (4)	0.025 (4)	0.021 (4)	0.013 (3)	0.009 (4)	0.009 (3)
C10	0.028 (4)	0.030 (5)	0.027 (4)	0.008 (3)	0.013 (4)	0.003 (3)
C11	0.044 (5)	0.036 (5)	0.027 (5)	0.017 (4)	0.016 (4)	−0.001 (4)
C12	0.037 (5)	0.035 (5)	0.024 (4)	0.024 (4)	0.007 (4)	0.004 (4)
C13	0.019 (4)	0.026 (4)	0.027 (4)	0.008 (3)	0.003 (3)	−0.002 (3)
C14	0.023 (4)	0.023 (4)	0.020 (4)	0.008 (3)	0.002 (3)	0.000 (3)
C15	0.017 (3)	0.018 (4)	0.017 (4)	0.005 (3)	0.007 (3)	−0.003 (3)
C16	0.015 (3)	0.015 (4)	0.021 (4)	0.003 (3)	0.003 (3)	0.003 (3)
C17	0.013 (3)	0.024 (4)	0.021 (4)	0.002 (3)	0.005 (3)	−0.002 (3)
C18	0.022 (4)	0.017 (4)	0.019 (4)	0.000 (3)	0.007 (3)	−0.004 (3)
C19	0.016 (3)	0.027 (4)	0.021 (4)	0.006 (3)	0.012 (3)	0.004 (3)
C20	0.013 (3)	0.034 (4)	0.019 (4)	0.007 (3)	0.006 (3)	0.000 (3)
C21	0.017 (4)	0.021 (4)	0.018 (4)	0.001 (3)	0.005 (3)	−0.002 (3)
C22	0.024 (4)	0.035 (5)	0.028 (4)	0.012 (3)	0.006 (4)	−0.004 (3)
C23	0.023 (4)	0.023 (4)	0.030 (4)	0.004 (3)	0.009 (4)	−0.003 (3)
C24	0.032 (4)	0.030 (5)	0.027 (4)	0.009 (3)	0.011 (4)	0.010 (3)
C25	0.029 (4)	0.020 (4)	0.018 (4)	0.002 (3)	0.002 (3)	−0.004 (3)
C26	0.031 (4)	0.039 (5)	0.030 (5)	0.010 (4)	0.021 (4)	−0.001 (4)
C27	0.030 (4)	0.033 (5)	0.032 (5)	0.016 (3)	0.013 (4)	0.009 (4)
C28	0.039 (5)	0.028 (5)	0.036 (5)	0.015 (4)	0.018 (4)	0.012 (4)
O1W	0.072 (5)	0.078 (5)	0.074 (5)	0.015 (5)	0.014 (5)	0.005 (4)

### Geometric parameters (Å, º)

**Table:**

S4—O14	1.493 (4)	O3—N2	1.220 (7)
S4—C29	1.766 (6)	O4—C9	1.301 (8)
S4—C30	1.773 (6)	O5—N4	1.227 (7)
C29—H29A	0.9800	O6—N4	1.230 (7)
C29—H29B	0.9800	O7—C16	1.445 (7)
C29—H29C	0.9800	O8—C17	1.413 (7)
C30—H30A	0.9800	O9—C20	1.417 (7)
C30—H30B	0.9800	O10—C21	1.419 (7)
C30—H30C	0.9800	N1—C7	1.281 (7)
S5—O15	1.494 (5)	N1—C15	1.499 (7)
S5—C31	1.768 (6)	N2—C5	1.451 (8)
S5—C32	1.778 (6)	N3—C14	1.280 (8)
C31—H31A	0.9800	N3—C19	1.496 (7)
C31—H31B	0.9800	N4—C12	1.437 (9)
C31—H31C	0.9800	C1—C2	1.437 (8)
C32—H32A	0.9800	C1—C6	1.397 (9)
C32—H32B	0.9800	C1—C7	1.443 (8)
C32—H32C	0.9800	C2—C3	1.412 (9)
S6A—O16A	1.488 (6)	C3—H3	0.9500
S6A—C33A	1.752 (8)	C3—C4	1.355 (8)
S6A—C34A	1.771 (9)	C4—H4	0.9500
C33A—H33A	0.9800	C4—C5	1.407 (9)
C33A—H33B	0.9800	C5—C6	1.377 (9)
C33A—H33C	0.9800	C6—H6	0.9500
C34A—H34A	0.9800	C7—H7	0.9500
C34A—H34B	0.9800	C8—C9	1.426 (9)
C34A—H34C	0.9800	C8—C13	1.395 (9)
S6B—O16B	1.495 (9)	C8—C14	1.469 (8)
S6B—C33B	1.742 (9)	C9—C10	1.411 (8)
S6B—C34B	1.775 (9)	C10—H10	0.9500
S6B—O17	2.025 (16)	C10—C11	1.355 (9)
C33B—H33D	0.9800	C11—H11	0.9500
C33B—H33E	0.9801	C11—C12	1.413 (10)
C33B—H33F	0.9801	C12—C13	1.375 (9)
C33B—O17	0.84 (3)	C13—H13	0.9500
C34B—H34D	0.9800	C14—H14	0.9500
C34B—H34E	0.9800	C15—C16	1.531 (8)
C34B—H34F	0.9800	C15—C17	1.524 (9)
S7—O17	1.501 (7)	C15—C18	1.517 (8)
S7—C35	1.762 (8)	C16—H16A	0.9900
S7—C36	1.776 (8)	C16—H16B	0.9900
C35—H35A	0.9800	C17—H17A	0.9900
C35—H35B	0.9800	C17—H17B	0.9900
C35—H35C	0.9800	C18—H18A	0.9800
C36—H36A	0.9800	C18—H18B	0.9800
C36—H36B	0.9800	C18—H18C	0.9800
C36—H36C	0.9800	C19—C20	1.518 (9)
Fe1—Cl1	2.378 (2)	C19—C21	1.520 (8)
Fe1—O1	1.924 (4)	C19—C22	1.540 (9)
Fe1—O7	2.038 (4)	C20—H20A	0.9900
Fe1—O9	1.989 (4)	C20—H20B	0.9900
Fe1—O11	2.018 (4)	C21—H21A	0.9900
Fe1—N1	2.133 (5)	C21—H21B	0.9900
Fe2—Cl2	2.3520 (18)	C22—H22A	0.9800
Fe2—O7	2.025 (4)	C22—H22B	0.9800
Fe2—O8	1.986 (4)	C22—H22C	0.9800
Fe2—O9	1.995 (4)	C23—H23A	0.9800
Fe2—O10	2.019 (5)	C23—H23B	0.9800
Fe2—O12	2.047 (4)	C23—H23C	0.9800
Fe3—Cl3	2.3319 (19)	C24—H24A	0.9800
Fe3—O4	1.948 (4)	C24—H24B	0.9800
Fe3—O8	1.971 (5)	C24—H24C	0.9800
Fe3—O10	2.036 (4)	C25—H25A	0.9800
Fe3—O13	2.045 (5)	C25—H25B	0.9800
Fe3—N3	2.158 (5)	C25—H25C	0.9800
S1—O11	1.531 (5)	C26—H26A	0.9800
S1—C23	1.776 (6)	C26—H26B	0.9800
S1—C24	1.773 (6)	C26—H26C	0.9800
S2—O12	1.537 (4)	C27—H27A	0.9800
S2—C25	1.773 (7)	C27—H27B	0.9800
S2—C26	1.786 (6)	C27—H27C	0.9800
S3—O13	1.542 (4)	C28—H28A	0.9800
S3—C27	1.789 (7)	C28—H28B	0.9800
S3—C28	1.776 (6)	C28—H28C	0.9800
O1—C2	1.308 (7)	O1W—H1WA	0.8698
O2—N2	1.235 (7)	O1W—H1WB	0.8700
			
O14—S4—C29	107.0 (3)	C20—O9—Fe1	126.5 (4)
O14—S4—C30	106.7 (3)	C20—O9—Fe2	128.8 (4)
C29—S4—C30	96.9 (4)	Fe2—O10—Fe3	101.55 (18)
S4—C29—H29A	109.5	C21—O10—Fe2	118.4 (4)
S4—C29—H29B	109.5	C21—O10—Fe3	111.4 (4)
S4—C29—H29C	109.5	S1—O11—Fe1	126.3 (3)
H29A—C29—H29B	109.5	S2—O12—Fe2	125.6 (2)
H29A—C29—H29C	109.5	S3—O13—Fe3	124.4 (3)
H29B—C29—H29C	109.5	C7—N1—Fe1	125.9 (4)
S4—C30—H30A	109.5	C7—N1—C15	118.9 (5)
S4—C30—H30B	109.5	C15—N1—Fe1	115.1 (4)
S4—C30—H30C	109.5	O2—N2—C5	118.9 (6)
H30A—C30—H30B	109.5	O3—N2—O2	122.7 (6)
H30A—C30—H30C	109.5	O3—N2—C5	118.5 (6)
H30B—C30—H30C	109.5	C14—N3—Fe3	127.2 (4)
O15—S5—C31	107.3 (3)	C14—N3—C19	118.3 (5)
O15—S5—C32	106.2 (3)	C19—N3—Fe3	114.4 (4)
C31—S5—C32	97.8 (3)	O5—N4—O6	122.9 (6)
S5—C31—H31A	109.5	O5—N4—C12	119.0 (6)
S5—C31—H31B	109.5	O6—N4—C12	118.2 (6)
S5—C31—H31C	109.5	C2—C1—C7	123.8 (6)
H31A—C31—H31B	109.5	C6—C1—C2	118.4 (6)
H31A—C31—H31C	109.5	C6—C1—C7	117.8 (6)
H31B—C31—H31C	109.5	O1—C2—C1	121.4 (6)
S5—C32—H32A	109.5	O1—C2—C3	120.0 (6)
S5—C32—H32B	109.5	C3—C2—C1	118.6 (6)
S5—C32—H32C	109.5	C2—C3—H3	119.1
H32A—C32—H32B	109.5	C4—C3—C2	121.8 (6)
H32A—C32—H32C	109.5	C4—C3—H3	119.1
H32B—C32—H32C	109.5	C3—C4—H4	120.2
O16A—S6A—C33A	106.6 (8)	C3—C4—C5	119.6 (7)
O16A—S6A—C34A	109.9 (8)	C5—C4—H4	120.2
C33A—S6A—C34A	97.6 (8)	C4—C5—N2	119.7 (6)
S6A—C33A—H33A	109.5	C6—C5—N2	119.7 (6)
S6A—C33A—H33B	109.5	C6—C5—C4	120.6 (6)
S6A—C33A—H33C	109.5	C1—C6—H6	119.5
H33A—C33A—H33B	109.5	C5—C6—C1	121.1 (6)
H33A—C33A—H33C	109.5	C5—C6—H6	119.5
H33B—C33A—H33C	109.5	N1—C7—C1	125.9 (6)
S6A—C34A—H34A	109.5	N1—C7—H7	117.1
S6A—C34A—H34B	109.5	C1—C7—H7	117.1
S6A—C34A—H34C	109.5	C9—C8—C14	122.6 (6)
H34A—C34A—H34B	109.5	C13—C8—C9	120.4 (6)
H34A—C34A—H34C	109.5	C13—C8—C14	117.0 (6)
H34B—C34A—H34C	109.5	O4—C9—C8	123.9 (6)
O16B—S6B—C33B	109.3 (16)	O4—C9—C10	119.0 (6)
O16B—S6B—C34B	105.2 (18)	C10—C9—C8	117.1 (6)
O16B—S6B—O17	126.1 (17)	C9—C10—H10	119.0
C33B—S6B—C34B	99.5 (14)	C11—C10—C9	122.1 (6)
C33B—S6B—O17	24.4 (10)	C11—C10—H10	119.0
C34B—S6B—O17	107.9 (16)	C10—C11—H11	119.9
S6B—C33B—H33D	109.5	C10—C11—C12	120.2 (6)
S6B—C33B—H33E	109.4	C12—C11—H11	119.9
S6B—C33B—H33F	109.6	C11—C12—N4	120.4 (6)
H33D—C33B—H33E	109.5	C13—C12—N4	120.0 (6)
H33D—C33B—H33F	109.5	C13—C12—C11	119.6 (7)
H33E—C33B—H33F	109.4	C8—C13—H13	119.7
O17—C33B—S6B	96.9 (18)	C12—C13—C8	120.5 (6)
O17—C33B—H33D	123.1	C12—C13—H13	119.7
O17—C33B—H33E	107.5	N3—C14—C8	125.3 (6)
O17—C33B—H33F	15.2	N3—C14—H14	117.3
S6B—C34B—H34D	109.5	C8—C14—H14	117.3
S6B—C34B—H34E	109.5	N1—C15—C16	105.1 (5)
S6B—C34B—H34F	109.5	N1—C15—C17	107.5 (5)
H34D—C34B—H34E	109.5	N1—C15—C18	114.4 (5)
H34D—C34B—H34F	109.5	C17—C15—C16	111.2 (5)
H34E—C34B—H34F	109.5	C18—C15—C16	109.0 (6)
O17—S7—C35	106.5 (7)	C18—C15—C17	109.5 (5)
O17—S7—C36	105.2 (7)	O7—C16—C15	111.2 (5)
C35—S7—C36	98.1 (8)	O7—C16—H16A	109.4
C33B—O17—S6B	58.6 (12)	O7—C16—H16B	109.4
C33B—O17—S7	169.1 (17)	C15—C16—H16A	109.4
S7—O17—S6B	110.7 (6)	C15—C16—H16B	109.4
S7—C35—H35A	109.5	H16A—C16—H16B	108.0
S7—C35—H35B	109.5	O8—C17—C15	112.7 (5)
S7—C35—H35C	109.5	O8—C17—H17A	109.0
H35A—C35—H35B	109.5	O8—C17—H17B	109.0
H35A—C35—H35C	109.5	C15—C17—H17A	109.0
H35B—C35—H35C	109.5	C15—C17—H17B	109.0
S7—C36—H36A	109.5	H17A—C17—H17B	107.8
S7—C36—H36B	109.5	C15—C18—H18A	109.5
S7—C36—H36C	109.5	C15—C18—H18B	109.5
H36A—C36—H36B	109.5	C15—C18—H18C	109.5
H36A—C36—H36C	109.5	H18A—C18—H18B	109.5
H36B—C36—H36C	109.5	H18A—C18—H18C	109.5
O1—Fe1—Cl1	97.77 (15)	H18B—C18—H18C	109.5
O1—Fe1—O7	160.59 (18)	N3—C19—C20	108.7 (5)
O1—Fe1—O9	93.60 (19)	N3—C19—C21	105.5 (5)
O1—Fe1—O11	91.01 (18)	N3—C19—C22	111.9 (5)
O1—Fe1—N1	86.88 (18)	C20—C19—C21	111.3 (6)
O7—Fe1—Cl1	94.34 (13)	C20—C19—C22	109.9 (5)
O7—Fe1—N1	78.53 (17)	C21—C19—C22	109.4 (5)
O9—Fe1—Cl1	167.12 (14)	O9—C20—C19	111.8 (5)
O9—Fe1—O7	76.39 (17)	O9—C20—H20A	109.3
O9—Fe1—O11	86.41 (17)	O9—C20—H20B	109.3
O9—Fe1—N1	99.18 (18)	C19—C20—H20A	109.3
O11—Fe1—Cl1	87.35 (14)	C19—C20—H20B	109.3
O11—Fe1—O7	104.69 (17)	H20A—C20—H20B	107.9
O11—Fe1—N1	174.13 (19)	O10—C21—C19	110.6 (5)
N1—Fe1—Cl1	87.51 (15)	O10—C21—H21A	109.5
O7—Fe2—Cl2	96.05 (13)	O10—C21—H21B	109.5
O7—Fe2—O12	97.47 (17)	C19—C21—H21A	109.5
O8—Fe2—Cl2	92.49 (12)	C19—C21—H21B	109.5
O8—Fe2—O7	90.10 (17)	H21A—C21—H21B	108.1
O8—Fe2—O9	89.57 (17)	C19—C22—H22A	109.5
O8—Fe2—O10	76.59 (17)	C19—C22—H22B	109.5
O8—Fe2—O12	171.43 (19)	C19—C22—H22C	109.5
O9—Fe2—Cl2	172.35 (14)	H22A—C22—H22B	109.5
O9—Fe2—O7	76.56 (17)	H22A—C22—H22C	109.5
O9—Fe2—O10	89.12 (18)	H22B—C22—H22C	109.5
O9—Fe2—O12	88.28 (16)	S1—C23—H23A	109.5
O10—Fe2—Cl2	98.52 (13)	S1—C23—H23B	109.5
O10—Fe2—O7	160.60 (17)	S1—C23—H23C	109.5
O10—Fe2—O12	95.08 (17)	H23A—C23—H23B	109.5
O12—Fe2—Cl2	90.72 (13)	H23A—C23—H23C	109.5
O4—Fe3—Cl3	96.40 (13)	H23B—C23—H23C	109.5
O4—Fe3—O8	93.36 (19)	S1—C24—H24A	109.5
O4—Fe3—O10	159.40 (18)	S1—C24—H24B	109.5
O4—Fe3—O13	96.78 (19)	S1—C24—H24C	109.5
O4—Fe3—N3	86.10 (18)	H24A—C24—H24B	109.5
O8—Fe3—Cl3	91.48 (13)	H24A—C24—H24C	109.5
O8—Fe3—O10	76.50 (17)	H24B—C24—H24C	109.5
O8—Fe3—O13	169.61 (17)	S2—C25—H25A	109.5
O8—Fe3—N3	95.57 (19)	S2—C25—H25B	109.5
O10—Fe3—Cl3	101.68 (13)	S2—C25—H25C	109.5
O10—Fe3—O13	93.15 (18)	H25A—C25—H25B	109.5
O10—Fe3—N3	77.26 (18)	H25A—C25—H25C	109.5
O13—Fe3—Cl3	89.66 (13)	H25B—C25—H25C	109.5
O13—Fe3—N3	82.88 (19)	S2—C26—H26A	109.5
N3—Fe3—Cl3	172.37 (16)	S2—C26—H26B	109.5
O11—S1—C23	102.8 (3)	S2—C26—H26C	109.5
O11—S1—C24	104.3 (3)	H26A—C26—H26B	109.5
C24—S1—C23	99.6 (3)	H26A—C26—H26C	109.5
O12—S2—C25	107.6 (3)	H26B—C26—H26C	109.5
O12—S2—C26	104.2 (3)	S3—C27—H27A	109.5
C25—S2—C26	97.6 (3)	S3—C27—H27B	109.5
O13—S3—C27	103.4 (3)	S3—C27—H27C	109.5
O13—S3—C28	104.6 (3)	H27A—C27—H27B	109.5
C28—S3—C27	99.6 (3)	H27A—C27—H27C	109.5
C2—O1—Fe1	134.9 (4)	H27B—C27—H27C	109.5
C9—O4—Fe3	134.7 (4)	S3—C28—H28A	109.5
Fe2—O7—Fe1	101.6 (2)	S3—C28—H28B	109.5
C16—O7—Fe1	110.6 (3)	S3—C28—H28C	109.5
C16—O7—Fe2	116.5 (3)	H28A—C28—H28B	109.5
Fe3—O8—Fe2	105.10 (19)	H28A—C28—H28C	109.5
C17—O8—Fe2	127.7 (4)	H28B—C28—H28C	109.5
C17—O8—Fe3	126.1 (4)	H1WA—O1W—H1WB	109.6
Fe1—O9—Fe2	104.4 (2)		
			
S6B—C33B—O17—S7	−10 (17)	C2—C3—C4—C5	1.3 (11)
O16B—S6B—C33B—O17	−137 (4)	C3—C4—C5—N2	−179.0 (6)
C34B—S6B—C33B—O17	113 (3)	C3—C4—C5—C6	−1.0 (11)
C35—S7—O17—S6B	−49.1 (10)	C4—C5—C6—C1	0.1 (11)
C35—S7—O17—C33B	−40 (16)	C6—C1—C2—O1	−178.5 (6)
C36—S7—O17—S6B	54.3 (9)	C6—C1—C2—C3	−0.1 (10)
C36—S7—O17—C33B	63 (16)	C6—C1—C7—N1	175.3 (6)
Fe1—O1—C2—C1	8.6 (10)	C7—N1—C15—C16	162.4 (6)
Fe1—O1—C2—C3	−169.8 (5)	C7—N1—C15—C17	−79.0 (7)
Fe1—O7—C16—C15	−49.5 (6)	C7—N1—C15—C18	42.8 (8)
Fe1—O9—C20—C19	144.7 (4)	C7—C1—C2—O1	3.5 (11)
Fe1—N1—C7—C1	−1.0 (10)	C7—C1—C2—C3	−178.1 (6)
Fe1—N1—C15—C16	−17.0 (6)	C7—C1—C6—C5	178.5 (6)
Fe1—N1—C15—C17	101.6 (5)	C8—C9—C10—C11	−2.6 (11)
Fe1—N1—C15—C18	−136.5 (5)	C9—C8—C13—C12	−1.3 (11)
Fe2—O7—C16—C15	65.7 (5)	C9—C8—C14—N3	−1.5 (11)
Fe2—O8—C17—C15	−44.0 (7)	C9—C10—C11—C12	−0.3 (12)
Fe2—O9—C20—C19	−41.6 (7)	C10—C11—C12—N4	−176.2 (7)
Fe2—O10—C21—C19	65.3 (6)	C10—C11—C12—C13	2.6 (12)
Fe3—O4—C9—C8	−5.7 (11)	C11—C12—C13—C8	−1.7 (11)
Fe3—O4—C9—C10	175.3 (5)	C13—C8—C9—O4	−175.6 (7)
Fe3—O8—C17—C15	150.0 (4)	C13—C8—C9—C10	3.4 (10)
Fe3—O10—C21—C19	−51.8 (6)	C13—C8—C14—N3	179.0 (7)
Fe3—N3—C14—C8	−1.2 (10)	C14—N3—C19—C20	−72.8 (7)
Fe3—N3—C19—C20	103.8 (5)	C14—N3—C19—C21	167.7 (6)
Fe3—N3—C19—C21	−15.6 (6)	C14—N3—C19—C22	48.8 (8)
Fe3—N3—C19—C22	−134.6 (5)	C14—C8—C9—O4	4.9 (11)
O1—C2—C3—C4	177.6 (6)	C14—C8—C9—C10	−176.0 (6)
O2—N2—C5—C4	1.0 (10)	C14—C8—C13—C12	178.2 (7)
O2—N2—C5—C6	−177.0 (7)	C15—N1—C7—C1	179.8 (6)
O3—N2—C5—C4	−178.9 (7)	C16—C15—C17—O8	59.6 (7)
O3—N2—C5—C6	3.0 (10)	C17—C15—C16—O7	−74.2 (6)
O4—C9—C10—C11	176.5 (7)	C18—C15—C16—O7	165.0 (5)
O5—N4—C12—C11	−172.6 (7)	C18—C15—C17—O8	−179.8 (5)
O5—N4—C12—C13	8.6 (11)	C19—N3—C14—C8	174.9 (6)
O6—N4—C12—C11	7.0 (11)	C20—C19—C21—O10	−75.6 (6)
O6—N4—C12—C13	−171.8 (7)	C21—C19—C20—O9	60.1 (6)
N1—C15—C16—O7	41.9 (6)	C22—C19—C20—O9	−178.5 (5)
N1—C15—C17—O8	−54.9 (6)	C22—C19—C21—O10	162.7 (5)
N2—C5—C6—C1	178.1 (6)	C23—S1—O11—Fe1	−117.7 (3)
N3—C19—C20—O9	−55.7 (7)	C24—S1—O11—Fe1	138.7 (3)
N3—C19—C21—O10	42.2 (7)	C25—S2—O12—Fe2	100.0 (4)
N4—C12—C13—C8	177.1 (7)	C26—S2—O12—Fe2	−157.1 (3)
C1—C2—C3—C4	−0.8 (11)	C27—S3—O13—Fe3	154.6 (3)
C2—C1—C6—C5	0.4 (10)	C28—S3—O13—Fe3	−101.6 (4)
C2—C1—C7—N1	−6.7 (11)		

### Hydrogen-bond geometry (Å, º)

**Table:**

*D*—H···*A*	*D*—H	H···*A*	*D*···*A*	*D*—H···*A*
C30—H30*A*···Cl2^i^	0.98	2.75	3.689 (7)	161
C32—H32*A*···O6	0.98	2.60	3.397 (9)	138
C32—H32*B*···Cl1^ii^	0.98	2.71	3.625 (6)	155
C32—H32*C*···Cl3^iii^	0.98	2.82	3.659 (7)	144
C34*A*—H34*A*···O4^iv^	0.98	2.52	3.47 (2)	164
C33*B*—H33*D*···O2^v^	0.98	2.35	3.06 (3)	128
C33*B*—H33*D*···N2^v^	0.98	2.26	3.08 (3)	141
C34*B*—H34*D*···O2^v^	0.98	2.58	3.19 (4)	120
C3—H3···O16*A*	0.95	2.60	3.391 (9)	141
C7—H7···O3^ii^	0.95	2.40	3.325 (8)	164
C23—H23*C*···O12	0.98	2.43	3.377 (8)	163
C25—H25*A*···Cl2	0.98	2.80	3.524 (6)	131
C25—H25*B*···O10^vi^	0.98	2.39	3.303 (7)	155
C26—H26*C*···O1*W*	0.98	2.55	3.393 (10)	144
C27—H27*A*···O14	0.98	2.27	3.243 (8)	171
O1*W*—H1*WA*···O15^vii^	0.87	2.12	2.949 (8)	158

Symmetry codes: (i) *x*+1, *y*, *z*; (ii) −*x*, −*y*+1, −*z*+1; (iii) −*x*, −*y*, −*z*+1; (iv) *x*+1, *y*+1, *z*; (v) −*x*+1, −*y*+2, −*z*+1; (vi) −*x*, −*y*, −*z*; (vii) *x*, *y*, *z*−1.
